# NaCl Effects on *In Vitro* Germination and Growth of Some Senegalese Cowpea (*Vigna unguiculata* (L.) Walp.) Cultivars

**DOI:** 10.5402/2013/382417

**Published:** 2013-07-25

**Authors:** Mahamadou Thiam, Antony Champion, Diaga Diouf, Mame Ourèye SY

**Affiliations:** ^1^Laboratoire Campus de Biotechnologies Végétales (LCBV), Département de Biologie Végétale, Faculté des Sciences et Techniques, Université Cheikh Anta Diop de Dakar, BP 5005, Dakar, Senegal; ^2^Laboratoire Mixte International Adaptation des Plantes et microorganismes associés aux Stress Environnementaux (LAPSE), LCM, Centre de Recherche de Bel Air, BP 1386, Dakar 18524, Senegal; ^3^Institut de Recherche pour le Développement (IRD), UMR DIADE, 911 avenue Agropolis, BP 64501, 34394 Montpellier Cedex 5, France

## Abstract

Cowpea (*Vigna unguiculata* (L.) Walp.) is one of the most important grain legumes in sub-Saharian regions. It contributes to man food security by providing a protein-rich diet. However, its production is limited by abiotic stresses such as salinity. This study aims to evaluate the salt tolerance of 15 cowpea cultivars, at germination stage. The seed germination process consisted of sowing them in agarified water (8 g·L^−1^) supplemented with 6 different concentrations of NaCl (0, 10, 50, 100, 150, and 200 mM). Results highlighted that high salt concentrations drastically reduced germination and significantly delayed the process for all varieties. A cowpea varietal effect towards the salt tolerance was noticed. Genotypes Diongoma, 58-78, and 58-191 were more salt-tolerant cultivars while Mougne and Yacine were more salt-sensitive ones as confirmed in the three groups of the dendrogram. NaCl effects on the early vegetative growth of seedlings were assessed with a tolerant (58-191) and a susceptible (Yacine) cultivar. Morphological (length and dry biomass) and physiological (chlorophyll and proline contents) parameter measurements revealed a negative effect of high (NaCl). However, 58-191 was much more salt tolerant, and the chlorophyll and proline contents were higher than those of Yacine genotype at increasing salt concentrations.

## 1. Introduction

Cowpea (*Vigna unguiculata*, (L.) Walp.) is a tropical herbaceous leguminous plant belonging to the *Fabaceae *family. This species is one of the most important grain legume crops in the Sub-saharian regions of Africa because several parts such as dry or fresh seeds (23–32% of protein and 64% of carbohydrate contains), the immature pods, and the leaves are used for human consumption. In addition, dry seeds, pods, and the hay are used for animal feeding during the dry season [1]. For this purpose, cowpea is a valuable source of income for farmers and grain traders in many African countries [2–4]. 

In Senegal, the economic importance of cowpea is increasing [5] as it is one of the essential crops for rural population diet [6]. Its cultivation is often associated with cereals such as millet, sorghum, and maize [7] due to its ability to establish a nitrogen-fixing symbiosis with *Bradyrhizobium* and/or mycorrhiza leading to soil fertility improvement [8]. The total cultivated area worldwide is estimated around 9.8 million ha, with a total production of 3.9 million tons in 2004 [9]. Senegal is a major producer of cowpea in West Africa with an estimated area of 130,730 ha and an average production of 37,648 tons [10].

Salinity is one of the main constraints for agricultural productivity affecting almost 80 million hectares of arable lands worldwide (20% of arable and 50% of irrigated lands) in the arid and coastal regions [11, 12]. A soil is considered saline when its electrical conductivity is 4 dS·m^−1^, approximately 40 mM NaCl [13]. Salt stress is induced by a wide range of dissolved salts, but NaCl is the most widespread one which explains the intensive investigations carried out [13–16]. To enhance understanding of the mechanisms of tolerance in high salinity conditions, several studies have been performed during the last three decades on the cowpea cultivars collected worldwide. These investigations led to the conclusions that saline soils present unfavorable conditions for seed germination and plant growth, limiting agricultural production. Indeed, irrigation induces an accumulation of salt at soil surface [17], negatively affecting germination, plant stand, plant vegetative development, productivity, and yield of cowpea, and, at the worst cases, it causes plant death [18–23]. According to Hall and Frate [24], cowpea is more tolerant to salinity than maize but more sensitive to it unlike wheat, barley, sugar beet and cotton.

Physiological studies clearly indicated that the negative effects of NaCl salinity were responsible for the increase of Na^+^ toxic ion interfering with K^+^ uptake leading to the disrupt of stomatal regulation, necrosis, reduction of growth, and loss of yield whereas Cl^−^ induced chlorotic toxicity symptoms due to chlorophyll degradation [14, 15, 25, 26]. In mungbean, one of the most salt-tolerant varieties of beans, salt stress provokes decrease in seed germination, shoot and root lengths, fresh and seedling vigor, chlorophyll a, b, and carotenoids content [27, 28]. Moreover, it is well documented that salt stress induces a large production of reactive oxygen species (ROS) in the chloroplast and mitochondria leading to lipid peroxidation, membrane injury, protein degradation, and enzyme inactivation [29, 30]. The salt-tolerant plants developed an enzymatic system such as superoxide dismutase (SOD), ascorbate peroxidase (APX), catalase (CAT), and glutathione peroxidase (GPX) which are playing a great role in the mitigation and repairing of the damage caused by the ROS activities. On the other hand, salt stress can be overcome by high accumulation of osmoprotectants in the cytoplasm which are classified in four main groups like the N-containing compounds (i.e., proline and glycine betaïne), sugars (i.e., sucrose and raffinose), straight-chain polyhydric polyols such as mannitol and sorbitol, and cyclic polyhydric alcohols [31–33].

Proline accumulates glycophytes as well as in in halophytes to restore the osmotic balance between cytoplasm and vacuole [34]. Therefore, proline synthesis is an adaptive reaction taken by the plant to overcome salinity stress induction. 

The objectives of this work were to study the *in vitro* germination behavior and to evaluate the physiological responses of different cowpea cultivars, collected from the Senegalese germplasm, submitted to various NaCl salinity stress in order to select the tolerant varieties. 

## 2. Materials and Methods 

### 2.1. Plant Material

The cowpea cultivars were provided by the “Centre National de la Recherche Agricole, CNRA, ISRA” at Bambey (Senegal). These cultivars have been chosen due to their adaptation to the Senegalese agroecological conditions, the benefit of getting a high coefficient of multiplication of quality seeds, and their use by the local farmers [35, 36]. The denomination and the botanical characteristics of the fifteen (15) selected cultivars are listed in Table 1.

### 2.2. Seed Disinfection and Germination Screening

Seeds of cowpea cultivars were surface-sterilized with 70% alcohol, followed by a stirred batch of bleach (NaOCl, 8°chl) for 15 min and a 3-time washing with sterile distilled water. Then, they were soaked for 3 h in sterilized distilled water and aseptically germinated in jars filled with 50 mL of an 8 g·L^−1^ agarified solution. 

Germination experiments consisted of 15 cowpea cultivars subjected to 6 different concentrations of salinity (0, 10, 50, 100, 150, and 200 mM [NaCl]) incorporated in a 0.8% agarified medium and pH adjusted to 5.8 before autoclaving at 110°C during 20 min. For each concentration, 12 seeds were maintained in the salinized medium, and the germination process followed for 10 days. Each treatment consisted of 3 jars inoculated with 4 seeds per jar. Jars were incubated in a dark oven at 28 ± 1°C for 10 days. To avoid a fast osmotic stress and salt ionic toxicity [38], salinization begins gradually. At 0 mM NaCl, 72 seeds subdivided into 6 batches of 12 seeds were planted in jars with 4 seeds per jar and 3 jars per treatment. Then, after 48 h, the other batches (60 seeds) were transferred into jars containing 10 mM [NaCl]. After another 48 h, 48 seeds (i.e., 3 batches of 12 seeds) were transferred into jars containing 50 mM [NaCl]. The procedure was continued every 48 h by transferring then in a new and higher concentration of salt, with a final batch of 12 seeds transferred to jars filled with a 200 mM [NaCl] medium.

For each cultivar and each saline treatment, a daily count of germinated seeds was performed and translated into cumulative germination percentage. The breakthrough of the radicle from the seed coats was used as the criterion for germination [39]. The effect of NaCl was studied by measuring the final cumulative rate of germination. 

### 2.3. *In Vitro* Growth of Seedlings

The experiment consisted of 2 selected cowpea cultivars, Yacine (sensitive) and 58-191 (tolerant). These varieties have been chosen because Yacine is a new popularized and improved variety while the 58-191 landrace is well adopted and appreciated by local farmers. They were submitted to 4 different doses of salt (0, 50, 100, 200 mM). A batch of 60 seedlings previously germinated, at a 2-leaf stage growth and carrying at least a 2 cm long root system, were individually transferred to a Gibson's glass tube (22 × 150 mm) filled with a MS liquid medium [40] and supplemented with 0, 50, 100, or 200 mM of NaCl. The aboveground part of the plants emerged outside the tube whereas the roots were directly in contact with the liquid medium at pH 5.8. To avoid mineral toxicity, the MS liquid media was renewed every week. Seedlings were incubated in a growth chamber at 28 ± 1°C, with a 16 h light/8 h night photoperiod and a light intensity provided by a synthetically active radiation of 83.33 *μ*E·m^−1^·s^−1^. To assess the saline stress, growth of a 12 cowpea plants batch for each cultivar was followed for 16 days by measuring the morphological and physiological parameters every 4 days. Each treatment consisted of 12 test tubes with 1 plant per Gibson's tube. 

Salinization began gradually 4 days after transplanting the plants in Gibson's glass tubes and continue up to 16 consecutive days until the highest salt level was achieved.

After the application of high saline stress to both contrasting cultivars (58-191 and Yacine), the number of surviving plants was counted every 4 days during 16 days of culture. The survival rate was defined as the ratio of the number of surviving plants in each count on the number of seedlings initially transferred.

### 2.4. Growth of the Aerial and Root Parts and Biomass Determination

Morphological parameters such as the length of the aerial part (LAP) and the plant root system (LRS) were measured using a ruler. After measurement, each part was separate from each other, washed in deionized water, surface-wiped with blotting paper, and dried in an oven (Memmert) at 80°C during 7 days. The dried biomass was weighed separately using a Sartorius balance (accuracy: 0.01) or a Sartorius precision scale (accuracy: 0.0001) for small samples. 

### 2.5. Determination of Chlorophyll Content

The assay of chlorophylls a, b, and total (t) used was based on the Arnon's technique [41]. Fifty  (50) mg taken from the median third youngest leaves were crushed in 10 mL of acetone at 80%. The homogenates were centrifuged at 5000 rpm for 10 min at 4°C (Sigma 3–30K). The absorbance of chlorophyll (b) and (a) was measured with a spectrophotometer (GENEWAY) at 645 nm and 663 nm, respectively. Chlorophyll (a), (b), and total chlorophyll (t) contents were calculated according to the Arnon's formula [41].

### 2.6. Extraction and Determination of Proline Concentration

For proline extraction, a sample of 100 mg of fresh leaves was mixed with 2 mL of 40% of methanol (v/v), heated in a waterbath at 85°C for 1 h. After cooling, a mixture consisting of 1 mL of leave extract, 1 mL of a 2.5% of ninhydrin solution (p/v), and 1 mL of a combined reaction (distilled water, acid acetic, and orthophosphoric acid at a ratio of 3/7.5/2) was composed. The mixture was well shaked for few seconds and incubated in a waterbath at 100°C for 30 min. After an ice cooling period of 3 min, 5 mL of toluene was added to the mixture and vortexed again. The upper phase of the mixture was collected and dehydrated with a pinch of anhydrous sodium sulfate. Then, absorbances of leave samples was measured and calculated. Proline contents were measured by colorimetry method as described by Monneveux and Nemmar [42]. The amount of proline, on a fresh-matter basis of plant leaves subjected to salt stress, was determined according to a calibration straight graph constructed from a series of standard proline solutions. The optical density of all samples were measured with a spectrophotometer (Jenway, Genova), at 528 nm. Each measure was repeated three times to ensure reproducibility of results.

### 2.7. Statistical Analysis

The cowpea experiment was set up as a standard randomized design, with salt concentration chosen as a main factor variable and cowpea cultivar as the subfactor variable. The data were subjected to a multiple comparison of means and to variance analysis with two factors (cultivars × [NaCl]) by the test of Student-Newman-Keuls. Analyzes were carried out according to a general linear model by the program Sigma Stat. For *in vitro* growth of seedlings under salt stress, differences between means were compared using the Newman and Keuls test, and significance was determined at 95% confidence limits.

### 2.8. Multivariate Analysis

The statistical package ADE-4 coupled with a hierarchical cluster analysis was used to run a normalized analysis of principal component to cluster the varieties according to their similarity. NaCl concentrations were considered as variable, but the 15 cowpea varieties were projected in a plane including the two first axes. The cowpea varieties were grouped using an ascending hierarchical clustering (AHC). The classification was performed by using the coordinates of the individuals on the factorial axes as similarity matrix, the Euclidean distance, and the Ward method. The R (version R-2.9.0, ADE4 package) software [43] was used to generate a dendrogram. The similarities revealed ranged from 0 (high similarity) to 12 (low similarity).

## 3. Results

### 3.1. Effect of NaCl on *In Vitro* Germination

The cumulative and final germination rates are shown in Table 2. Most cultivars germinated at a rate of 100%, as the control groups at 10 and 50 mM [NaCl], except for Bambey 21, 58-53, Yacine, and Mougne cultivars which revealed a significant decrease in the germination rates of 75%, 25%, 25%, and 50%, respectively. The results showed that Mougne and Yacine cultivars had a greater sensitivity to salinity, their germination rate dropped significantly at 10 mM [NaCl], with respective rates of 75% and 50%. At 100 mM [NaCl], cultivars 58-184, 58-3, 58-191, Melakh and Diongoma retained their germination rate of 100%, while the others cultivars were affected with different behaviors. Germination of Yacine, Mougne, and 58-53 cultivars was completely inhibited. Cultivars 58-78, 58-57, and 58-74F had a relatively high percentage of germination (75%) while the germination rate of Bambey 21, Ndiaga aw, Ndiambour, and 58-80 was significantly lowered with values of 25%, 50%, 25%, and 50%, respectively. 150 mM of NaCl inhibited the germination of cultivars Ndiambour, Ndiaga Aw and Melakh. At 200 mM of NaCl, only six cultivars 58-3, 58-191, 58-78, 58-80, 58-57, 58-74F, and Diongoma could germinate at different rates. The highest germination rate (75%) was recorded with cultivars Diongoma and 58-78.

### 3.2. Genetic Relationship between Cultivars

**Figure 1:**
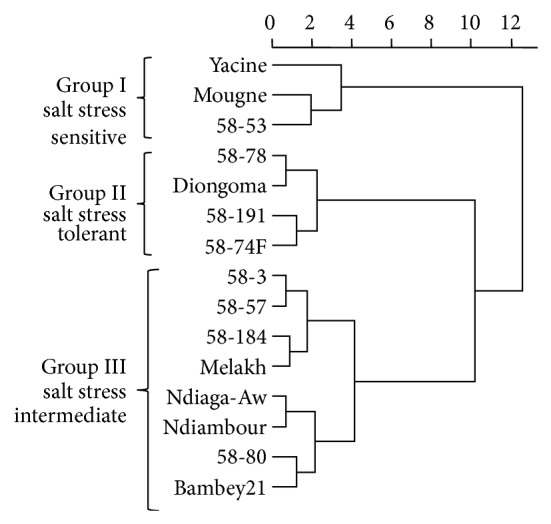
Dendrogram showing similarity between 15 varieties based on NaCl salinity stress.

The dendrogram in Figure 1 summarized the genetic relationship between the cowpea cultivars based on salinity stress. Cowpea cultivars can be divided into three clusters. The first group encompassed the NaCl salt-sensitive cultivars: Yacine, Mougne, and 58-53. The second cluster included two subgroups. The first subgroup was formed by Diongama and 58-78 while the second one encompassed the local and tolerant cultivars 58-191 and 58-74F. In the third group, 2 subgroups were also identified; each of them was encompassing 2 other subgroups. The first subgroup included the local cultivars 58-3, 58-57, 58-181 and the inbreed line Melakh. In the second subgroup, the local cultivar Ndiaga Aw and the inbreed line Ndiambour were clustering. In the other subgroup, the local cultivar 58-80 and the inbreed line Bambey 21 formed the same clade. 

### 3.3. Effect of NaCl on *In Vitro* Growth of Seedlings

#### 3.3.1. Survival Rates

**Figure 2:**
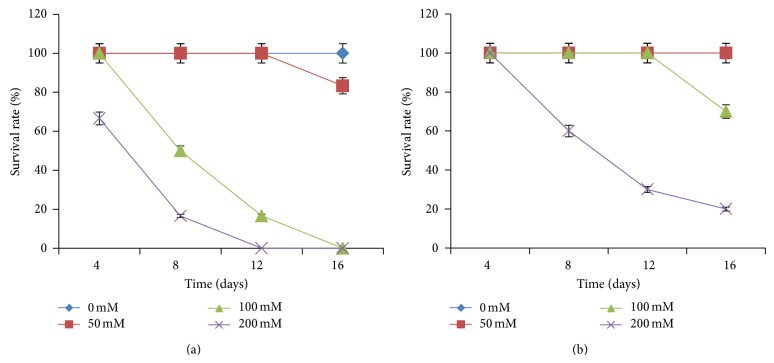
Evolution of survival rate of Yacine (a) and 58-191 (b) cultivars under NaCl treatments.

Figure 2 revealed that for control plants grown at 0 mM [NaCl], the survival rate (100%) did not vary during the experiment for both cultivars. The survival rate of plants (100%) grown in 50 mM [NaCl] did not change for the cultivar 58-191 during the first 12 days but started to decrease to 70% and to 83.3% for the cultivar Yacine at 16 days. At 100 mM [NaCl], the genotype Yacine showed a significant decrease in survival rate in the first days as soon as the seedlings were adapted to salinized media. This rate was equivalent to 50%, 20%, and 0%, respectively, after 8, 12, and 16 days. The survival rate decreased for both cultivars at 200 mM [NaCl]. However, the cultivar Yacine was more affected. Its survival rate was equal to 0% after 12 days of culture against 30% and 20%, respectively, after 12 and 16 days for 58-191. These results showed that, at this stage of development, the cultivar 58-191 was more tolerant to salinity compared to Yacine. 

#### 3.3.2. Impact of Salinity on the Length of the Aerial and Root Parts

Table 3 shows the variation of the aerial length parts of two contrasting cultivars (Yacine and 58-191) depending on the concentration of NaCl in the media. At 50 mM and 100 mM [NaCl], growth in height of the cultivar 58-191 was not affected whereas the length of the aerial part in Yacine was reduced to 7.78 and 7.16 cm, respectively. However, the growth of both cultivars was negatively affected at 200 mM [NaCl]. A significant reduction of the aboveground length in both cultivars was noted with a decrease to 3.87 cm and 4 cm for Yacine and 58-191, respectively. 

Table 3 represents also the results of the cowpea cultivars behavior in terms of length growth of the root parts. Root length of Yacine cultivar was adversely affected with a significant reduction at 50 and 100 mM [NaCl] and reached 6.95 cm and 6.33 cm, respectively, while the cultivar 58-191 was not affected. Consequently, depending to the cultivars, high NaCl concentration affected significantly the root growth. The comparison of mean values revealed a very highly significant difference (*P* < 0.001) between treatments and between cultivars (Table 2). For 200 mM [NaCl], there was a significant negative effect for both genotypes (*P* < 0.01). 

#### 3.3.3. Biomass Determination

The variance analysis of the variable dry weight showed a significant difference between the different concentrations of NaCl. The ranking of means (Table 4) demonstrated, for the dry weight of the aboveground part, that a negative effect of different doses of [NaCl] (*P* < 0.001) among cultivars existed (*P* = 0.052). With regard to the root dry weight (RDW), there was a significant difference between treatments (Control, 50, 100, and 200 mM) in the cultivar 58-191 (*P* < 0.001). In Yacine cultivar, a significant difference between the control and 100 mM [NaCl] was noticed (*P* < 0.001), but no significant difference of RDW treated with 100 and 200 mM [NaCl] (*P* > 0.05) was revealed. In addition, the negative effect of 100 mM [NaCl] treatment was more pronounced in Yacine than in 58-191 cultivars. Indeed, in the cultivar Yacine, the dry aerial biomass decreased from 0.106 g for the control to 0.006 and 0.054 g, respectively, at 100 and 200 mM [NaCl]. On the other hand, with 58-191 cultivar, the aerial dry biomass declines from 0.096 g to 0.07 g at 100 mM [NaCl] and to 0.053 g at 200 mM [NaCl]. Salt-stressed seedlings exhibited similar trends for dry root biomass. Indeed, in Yacine, the root dry biomass for control plants was 0.092 g, and it decreased to 0.044 g and 0.024 g for 100 and 200 mM [NaCl], respectively. The dry root biomass of plants belonging to 58-191 cultivar was reduced by only 0.107 g to 0.070 g at 100 mM [NaCl] and to 0.025 g at 200 mM [NaCl]. 

#### 3.3.4. Impact of Salinity on Chlorophyll Content in Plant Leaves

The analysis of variance showed a significant negative effect of salt stress on the accumulation of total chlorophyll in both genotypes (Table 5). The comparison of mean total chlorophyll revealed that treatments with 50 and 100 mM [NaCl] induced a significant negative effect in the genotype Yacine but had no significant effect in the genotype 58-191. For the treatment at 200 mM [NaCl], there was a significant negative effect in both genotypes. Indeed, in Yacine cultivar, total chlorophyll content has decreased from 2.52 mg·g^−1^ fresh weight (FW) to 1.56 mg·g^−1^ FW and to 1.35 mg·g^−1^ FW at 100 and 200 mM [NaCl], respectively. With the genotype 58-191, total chlorophyll content was equivalent to 2.3 mg·g^−1^ FW in the control and plants grown under 100 mM [NaCl]. It tumbled down significantly to 1.61 mg·g^−1^ FW at 200 mM [NaCl].

The analysis of variance at a single classification criterion applied to the total chlorophyll content showed a very highly significant difference (*P* < 0.001). The Newman & Keuls test revealed three homogeneous clusters for Yacine cultivar and two groups for the cultivar 58-191. Group A represented the control plants, the group B represented plants submitted to moderate salt stress, and group C included the plants under severe saline treatments (200 mM [NaCl]). Furthermore, when salt stress was moderate (50 mM), chlorophyll a decreased slightly. However, when stress was high (100–200 mM [NaCl]), chlorophyll a decreased more significantly than chlorophyll b, specifically in Yacine cultivar.

#### 3.3.5. Impact of Salinity on Proline Content in Plant Leaves

**Figure 3:**
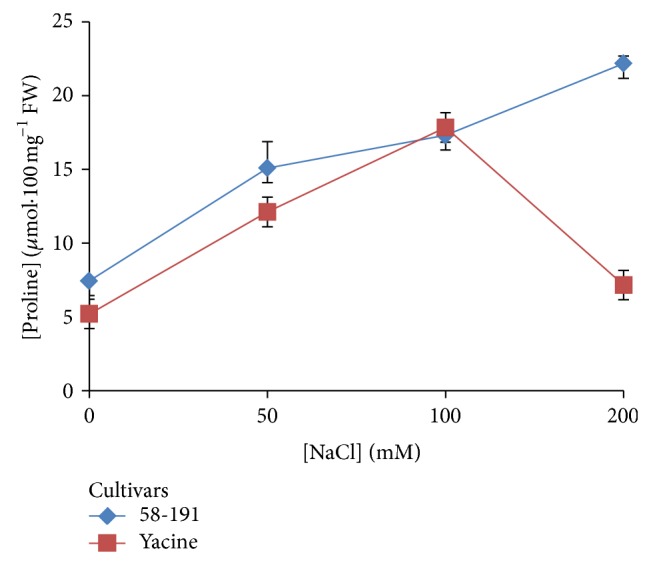
Variation of the proline contents of the different cultivars 58-191 and Yacine according to an increasing range of salt concentrations in the culture medium.

Absorbances obtained from leave samples were reported on the calibration curve which was used to determine their proline contents. This standard curve revealed a linear regression of proline contents with *R* ^2^ equivalent to 0.9965 (data not shown). Proline concentrations in both cultivars were low in media without NaCl (control plants) and increased as NaCl concentrations increased in the media up to 100 mM. Indeed, in Yacine cultivar, the lowest value (5.207 *μ*mol·100 mg^−1^ FW) was recorded while foliar samples collected from 58-191 cultivar had a proline content of 7.444 *μ*mol·100 mg^−1^ FW (Figure 3 and Table 6). The analysis of variance showed that this difference was significant at less than 5% (*P* < 0.001). At a salt-stress dose of 50 mM [NaCl], there was an increase of proline content in both cultivars with levels of 15.104 and 12.122 *μ*mol·100 mg^−1^ FW, respectively, for 58-191 and Yacine genotypes. The variance analysis revealed that these values were significantly different from those of the control ones. When the [NaCl] dose increased to 100 mM, there was also a significant increase (*P* < 0.05) of the proline content in both cultivars. However, the increase was slightly higher in the cultivar Yacine where it evolved from 12.122 to 17.846 *μ*mol·100 mg^−1^ FW whereas the increase in 58-191 was smaller, ranging from 15.104 to 17.313 *μ*mol·100 mg^−1^ FW. Additionally, analysis of variance revealed no significant difference between the two cultivars in 100 mM (*P* = 0.085). At 200 mM [NaCl], a drastic and significant decrease was noticed for Yacine cultivar; proline content was equivalent to 7.151 *μ*mol·100 mg^−1^ FW while for 58-191 cultivar, the proline content reached 22.193 *μ*mol·100 mg^−1^ FW. The analysis of variance confirmed a significant difference between the two cultivars at 200 mM [NaCl] at *P* < 0.001. 

## 4. Discussion

### 4.1. Germination

Germination or seedling establishment is a critical process in a plant's life, especially in the presence of adverse environmental factors [44]. For this purpose, fifteen genotypes of cowpea seeds were tested for salt tolerance, at the germination stage, in jars at different salinity levels (0, 10, 50, 100, 150, and 200 mM [NaCl]) in order to identify contrasted cultivars, that is, salttolerant versus sensitive. 

This study showed that salinity significantly affects germination of cowpea seeds and variability in behavior between different cultivars. Most cultivars germinate at low concentrations (10–50 mM). However, high concentrations of NaCl (100, 150, and 200 mM) resulted in significant reduction in the rate of seed germination for some cultivars and complete inhibition of germination for others. Inhibition of final germination rate for sensitive cultivars resulted from a difficulty of seed hydration due to high osmotic potential. This can be explained by the time required for seeds to implement mechanisms for adjusting their internal osmotic pressure. Thus, on the basis of this criterion, the cultivars Yacine, Mougne and 58-3, whose germination is significantly diminished after the first dose of salt (10 mM), are the most sensitive. Salt-tolerant cultivars are Diongoma, 58-184 and 58-191, whose germination rates were significantly reduced at 150 mM [NaCl]. These results corroborate those obtained by several authors on the effect of NaCl on germination of cowpea [18, 45, 46]. In addition to reducing the germination rate for sensitive cultivars (Yacine), salt stress also delays germination and slows its speed. Decrease observed may be due to the alteration of enzymes and hormones contained in the seeds [47] or to a problem of seed hydration due to a high osmotic potential which inhibits the emergence of the radicle off husks [48].

### 4.2. Genetic Relationship between Varieties Based on Salinity Stress

Based on Ward's distance, the cowpea varieties were clustered into 3 groups according to their sensitivity on salt stress during the germination stage. The varieties making up the group 1 were salt tolerant which included the inbred lines Yacine and Mougne, and the local variety 58-53. Mougne, and 58-53 cultivars are prostrate with bicolor white flowers, none photosensitive and sensitive to Amsacta. However, they differed in their seeds color which is white for 58-53 and gray for Mougne. The low number of shared agronomic characteristics should explain the 2% similarities reported on the dendrogram. The salt-tolerant group (group 2) included the local varieties except the inbred lines Diongoma which were not clustering with one of its parent 58-57. The results corroborate the findings of Bohnert et al. [49] suggesting that salinity tolerance is controlled by multiple genes. In contrast, genetic relationship based on microsatellite markers classified Diongoma in the same group as one of its genitor 58-57 [37]. In the dendrogram, the grouping of Diongoma and 58-78 with 1% of coefficient of similarity was in agreement with the number of agronomic characters shared between these varieties which was the none photoperiod sensitivity and the sensitivity to Amsacta. Moreover, the grouping of 58-74F with 58-191 in these studies was in agreement of the results reported by [37] using microsatellite markers, supporting the closest genetic base of salt tolerance between these varieties. The grouping of the landraces among the salt tolerant suggests that a genetic basis of NaCl salinity tolerance should exist in Senegalese germplasm. Ndiambour and one of its progenitor 58-57 were in the same group named the salt-tolerant intermediate as it was previously described on the data based on microsatellite markers [37]. In addition, this group is formed by landraces and some inbred lines resulting from a cross between the local varieties and those from International Institute of Tropical Agriculture (IITA) [50]. 

### 4.3. Effect of NaCl on *In Vitro* Growth of Seedlings

#### 4.3.1. Survival Rates

Plant survival is often chosen as the main criterion for salt tolerance in crop plants [51–54]. This study has shown that the survival of young cowpea plants depends on the cultivar, treatment severity, and duration of salt stress application. Thus, the cultivar 58-191 maintains a survival rate of 65% after two weeks of treatment with 100 mM [NaCl] and was classified as salt tolerant and Yacine cultivar as salt sensitive. Indeed, only 50% of the seedlings could survive after only 8 days of stress, and after two weeks of stress, no plants survived at 100 mM [NaCl]. These results are consistent with those found by Mezni et al. [55] in three alfalfa perennial cultivars and those obtained by Murillo-Amador et al. [19] on cowpea accessions of different origins.

#### 4.3.2. Morphological and Physiological Parameters

Our results have shown that salt has a negative effect on the growth of cowpea seedlings. Using various concentrations of NaCl, different behaviors depending on the cultivar were observed. Statistical analysis showed that 100 mM [NaCl] significantly slowed the growth in length of the aerial parts, mainly the hypocotyls and roots of cultivar Yacine while growth in length of 58-191 cultivar was not affected by this concentration. According to several authors, the salt stress significantly reduced the growth of roots and shoots, for both adult plants and seedlings [38, 56, 57]. 

The effect of NaCl on the growth of cowpea was morphologically reflected by a stunting of shoots and roots (data not shown). But the depressive effect of salt occurs mainly in young leaves than in roots, during the early vegetative stage of development. This difference in sensitivity between the organs of absorption and those of photosynthesis is characteristic of glycophytes [58, 59]. The poor development of these parts is due to the increase in osmotic pressure in the medium, ionic toxicity of sodium and chlorine to the roots, and nutritional imbalance of the plant caused by a lack of absorption and/or transport of nutrients to the stem [60]. 

The effect of salt stress was also evaluated on the basis of growth parameters such as biomass production. So, this effect on cowpea was also evident on the production of dry matter of the aerial and roots parts. The decrease in production of dry biomass is a classic response to salt stress and was previously used to evaluate the kinetic of the dry matter mass [61, 62].

Overall, the dry weight of aerial parts is more important for the cultivar Yacine than that of 58-191 which is tolerant genotype. But, for the cultivar Yacine, the ratio of root dry weight parts of the aerial parts was less than 1 and decreased sharply as the salt concentration increased. Conversely, for the cultivar 58-191, this ratio was greater than 1 for both control plants and those treated with 50 and 100 mM [NaCl]. Salt intake seems to induce in this cultivar, more tolerant to salt, an increase in dry weight of roots. But the difference between the two genotypes fades when the stress is equal to 200 mM [NaCl]. Our results confirm also those reported by Sanchez-blanco et al. [63], demonstrating that the decrease in the leaf dry weight of tomato is a consequence of salinity. LIyod et al. [64] showed that Na^+^ is much more responsible for the reduction in gas exchange and CO_2_ assimilation rates and, consequently, growth. 

In this work, the concentration of chlorophyll a was lower in genotype Yacine than in genotype 58-191, mostly at 100 mM [NaCl]. However, this difference is less obvious at 200 mM [NaCl] because of the significant reduction in chlorophyll a in both genotypes which can be justified by the large accumulation of Na^+^ and Cl^−^ ions as described by [65]. Total chlorophyll content decreases with increasing stress intensity in both cowpea cultivars according to what several authors have reported [66, 67]. Reduction of photosynthesis is largely due to stomatal closure and possibly reduced mesophyll conductance, that is, the chlorophyll parenchyma [68] caused by the loss of turgor and the disturbance of root signals [68–70].

The ratio chl.a/chl.b is also a good indicator of tolerance to water stress and is an important parameter to study the influence of salt stress as well. Chlorophyll a is much more sensitive to the action of abiotic stress than chlorophyll b. Under stress, this ratio decreased on plants of the sensitive cultivar Yacine. In fact, when NaCl concentrations increased, the ratio chl.a/chl.b decreased in Yacine cultivar. These results corroborate those reported by Guettouche [71] suggesting that the higher this parameter is, the greater varieties are tolerant to water deficit or saline stress. 

The determination of proline content accumulated and induced under stress is considered as one of the fast and efficient techniques for evaluating the salt tolerance in plants.

In our experiment, proline content increased progressively with the different concentrations of NaCl tested in the salt-tolerant cultivar 58-191, contributing to the regulation of osmotic pressure in the cell compartment. These results are in accordance with those of Camara et al. [56] which observed an increase in proline, glutamine, and other aminoacids in maize calli subjected to NaCl concentrations higher than 100 mmol·L^−1^. Both osmotica induced stomatal closure and accumulation of toxic levels of Na^+^ in the cell's cytosol under saline conditions which reduce a plant's capacity to fully utilize light absorbed by the photosynthetic pigments and leads to the formation of various reactive oxygen species [72]. The accumulation of proline resulted from the decrease of protein synthesis, conversion of glutamate to proline, and induced pH regulation [73, 74]. 

## 5. Conclusion

A great amount of literature described the effects of salinity in adult cowpea plants [75]. However, its effects during seed germination in different cultivars still remain less documented. Due to this fact, more studies on salt-stressed seed germination are necessary for the complete elucidation of its effect on cowpea germination and seedling development. This work shows that salt stress has, in all cowpea accessions tested, a depressive effect on seed germination and on all physiological and morphological parameters studied. However, it does not affect them in the same way depending on the intensity and the duration of stress and cultivar as well. The results suggest that cowpea plant is sensitive to NaCl at germination stage. At 150 mM (NaCl), the germination capacity of all cultivars is greatly reduced. In addition, an intraspecific quite important variability, in presence of the salt stress, was observed between the 15 cultivars as noticed in the dendrogram which revealed 3 main different groups related to the degree of tolerance to salt stress. Thus, in the germination stage, cultivars 58-3, Melakh, 58-191, 58-184, and Diongama are more tolerant to salinity, with a germination rate of 100% in the presence of 100 mM (NaCl) while cultivars Yacine, Mougne, and 58-53 whose germination was inhibited at the same concentration of salt are the most sensitive to salinity. The effects of salt stress on the growth of two contrasting cultivars at germination stage were also analyzed. Our results showed, for all growth parameters measured, a depressive effect of salt stress. However, among both cultivars, 58-191 was more tolerant than Yacine. This confirms the trend noted in the germination stage. Such variability can be used later in breeding programs associated with the identification of molecular markers linked to salt tolerance in cowpea. 

## Figures and Tables

**Table 1:** Agronomic characteristics of 15 cowpea accessions cultivated in Senegal [37].

Varieties	Pedigree	Growth habit	Flowers	Seeds	Sensitivity to day length	CAbMV	Bacterial	*Striga *	Amsacta	Aphids	Thrips	Bruchid
58-184	Local	Prostrate	Bicolor white	Gray	NP	—	—	—	S	—	—	—
58-191	Local	Prostrate	Bicolor white	Gray	NP	—	—	—	S	—	—	—
58-3	Local	Prostrate	Bicolor white	Violet	NP	—	—	—	S	—	—	—
58-53	Local	Prostrate	Bicolor white	White	NP	—	—	—	S	—	—	—
58-57	Local	Prostrate	Bicolor white	NP	NP	—	—	—	S	—	—	—
58-74F	Local	Semierect	Bicolor white	Gray-violet	NP	—	—	—	S	—	—	—
58-78	Local	Semierect	Bicolor white	White	NP	—	—	—	S	—	—	—
58-80	Local	Semierect	Purple	White-red	NP	—	—	—	S	—	—	—
Bambey 21	5/8 of 58-40 +1/4 of 66-74+s 1/8 of 58650	Erect	White	White		R	S	S	S	S	S	S
Melakh	IS86-292 × IT83s-742-13	Prostrate	White	White brown eyed	NP	R	R	S	S	R	S	S
Mougne	58-74 × Pout	Prostrate	Bicolor white	Gray	NP	S	R	S	S	S	S	S
Ndiaga aw	Local	Prostrate	Bicolor white	Red	NP	S	R	S	S	S	S	S
Ndiambour	58-41 × 58-57	Prostrate	Bicolor white	Cream beige eyed	NP	S	R	S	S	S	S	S
Diongoma	58-57 × IT81D-1137	Erect	White	White beige eyed	NP	R	R	R	S	S	S	S
Yacine	Ndiaga Aw × Melakh	Erect	White	Red	NP	R	R	S	S	R	S	S

NP: not photosensitive; R: resistant; S: sensitive; CAbMV: cowpea aphid-borne mosaic virus; —: no available data.

**Table 2:** Comparison of final germination rates (%) of 15 cowpea cultivars by Student-Newman-Keuls test at the threshold of 5%.

Cowpea cultivars	Final germination rates (%)					
	NaCl concentration (mM)					
	0	10	50	100	150	200
Diongoma	100a	100a	100a	100a	75b	75b
58-78	100a	100a	100a	75b	75b	75b
58-191	100a	100a	100a	100a	75b	50c
58-3	100a	100a	100a	100a	25d	25d
58-80	100a	100a	75b	50c	25d	25d
58-74F	100a	100a	100a	75b	75b	25d
58-57	100a	100a	100a	75b	25d	25d
58-184	100a	100a	100a	100a	25d	0e
Melakh	100a	100a	100a	100a	0e	0e
Ndiaga Aw	100a	100a	100a	50c	0e	0e
Ndiambour	100a	100a	100a	25d	0e	0e
Bambey 21	100a	100a	75b	25d	25d	0e
58-53	100a	100a	25d	0e	0e	0e
Mougne	100a	75b	25d	0e	0e	0e
Yacine	100a	50c	50c	0e	0e	0e

In lines, values followed by the same letter are not significantly different according to the Student-Newman-Keuls test (*P* < 0.05).

**Table 3:** Mean comparison of shoot and root part lengths by Student Newman-Keuls test at 5%.

Cultivars	NaCl treatment (mM)	LAP (cm)	LRS (cm)	TLP (cm)	LAP/LRS	LRS/LAP	(LAP/TLP)/100	(LRS/TLP)/100	Reduction rate LAP (%)	Reduction rate LRS (%)
Yacine	0	14.66a	9.5a	24.16a	1.54	0.64	60.67	39.32	—	—
Yacine	50	7.78b	6.95b	14.73b	1.12	0.89	52.81	47.18	−46.93	−26.84
Yacine	100	7.16b	6.33b	13.49b	1.13	0.88	53.07	46.92	−51.16	−33.37
Yacine	200	3.87c	2.37c	6.24c	1.63	0.61	62.01	37.98	−73.60	−75.05

58-191	0	18a	10.16a	28.16a	1.77	0.56	63.92	30.07	—	—
58-191	50	17.85a	10a	28a	1.80	0.55	63.75	35.71	0.83	−1.57
58-191	100	18a	9.95a	27.8a	1.79	0.55	64.74	35.79	0	−2.06
58-191	200	4b	2b	6b	2.00	0.13	66.66	33.33	−77.77	−80.31

For each cultivar, values in the same column followed by the same letter do not differ significantly between cultivars at 5% level. LAP: length of aerial part; LRS: length of root system; TLP: total length of Plant.

**Table 4:** Mean comparison of shoot and root parts dry weights by Student-Newman-Keuls test at 5%.

Cultivars	NaCl Treatment (mM)	ADW (g)	RDW (g)	DTBW (g)	RDW/ADW	(ADW/DTBW) ×100	(RDW/DTBW) ×100	Reduction rate ADW (%)	Reduction rate RDW (%)
Yacine	0	0.103a	0.092a	0.20a	0.893	52.82	47.17	—	—
Yacine	50	0.11a	0.046b	0.16ab	0.418	70.51	29.48	−19.41	−50.00
Yacine	100	0.06b	0.044b	0.10b	0.733	57.69	42.30	−41.74	−52.17
Yacine	200	0.054b	0.024b	0.08b	0.444	69.23	30.76	−47.57	−73.91

58-191	0	0.097a	0.107a	0.20a	1.103	47.54	52.45	—	—
58-191	50	0.096a	0.108a	0.20a	1.125	47.05	52.94	−1.03	+0.93
58-191	100	0.07b	0.07b	0.14b	1.000	50	50	−27.83	−34.58
58-191	200	0.053c	0.025c	0.078c	0.471	67.94	32.05	−45.36	−76.63

For each cultivar, values in the same column followed by the same letter do not differ significantly between cultivars at 5% level. ADW: aerial part dry weight; RDW: root dry weight; DTBW: Dry total biomass weight.

**Table 5:** Mean comparison of chlorophyll a, b, and total chlorophyll contents by the method of Student-Newman-Keuls at 5%.

Cultivars	NaCl treatments (mM)	Chlorophyll a (mg · g^−1^ FM)	Chlorophyll b (mg · g^−1^ FM)	Total Chlorophyll (mg · g^−1^ FM)	Chl a/Chl b	Homogeneous groups
Yacine	0	1.45a	1.07a	2.52a	1.35a	A
Yacine	50	0.75b	1.02a	1.77b	0.74b	B
Yacine	100	0.52c	1.04a	1.56c	0.50c	C
Yacine	200	0.43c	0.92a	1.35d	0.47c	C

58-191	0	1.56a	1.04a	2.30a	1.50a	A
58-191	50	0.75b	0.96a	2.47a	0.78b	B
58-191	100	1.44ab	0.93a	2.37a	1.55ab	B
58-191	200	0.67b	0.94a	1.61b	0.71b	B

For each cultivar, values in the same column followed by the same letter do not differ significantly between cultivars at the level of 5%.

**Table 6:** Mean comparison, by Student Newman-Keuls test at 5%, of proline contents determined among 58-191 and Yacine cultivars submitted to different salt stress conditions.

[NaCl] concentrations (mM)	[Proline] contents (µmol · 100 mg^−1^ FW)	
	Cultivars	
	58-191	Yacine
0	7.444aA	5.207aB
50	15.104bA	12.122bB
100	17.313cA	17.846cA
200	22.193dA	7.158dB

On the same column, values assigned to the same lowercase letter are not significantly different. On the same line, the values assigned to the same capital letter are not significantly different.

## References

[1] Chinma C. E., Alemede I. C., Emelife I. G. (2008). Physicochemical and functional properties of some Nigerian cowpea varieties. *Pakistan Journal of Nutrition*.

[2] Langyintuo A. S., Lowenberg-DeBoer J., Faye M. (2003). Cowpea supply and demand in West and Central Africa. *Field Crops Research*.

[3] Timko M. P., Ehlers J. D., Roberts P. A., Kole C. (2007). Cowpea. *Pulses, Sugar and Tuber Crops, Genome Mapping and Molecular Breeding in Plants*.

[4] Timko M. P., Singh B. B., Moore P. H., Ming R. (2008). Cowpea, a multifunctional legume. *Genomics of Tropical Crop Plants*.

[5] Boufroy E. (1994). *Analyse Éco-Physioloque Et Agronomique Des Perspectives D'Amélioration De La proDuction De Semences De Niébé Au Sénégal*.

[6] Cissé N., Hall A. E. (2003). *Traditional Cowpea in Senegal, A Case Study*.

[7] Rachie K. O., Roberts L. M. (1974). Grain legumes of the lowland tropics. *Advances in Agronomy*.

[8] Akinyele I. O., Onigbinde A. O., Hussain M. A., Omololu A. (1986). Physicochemical characteristics of 18 cultivars of Nigerian cowpeas (*V.unguiculata*) and their cooking properties. *Journal of Food Science*.

[9] FAOSTAT http://faostat.fao.org/.

[10] DSDIA/DAPS/MAE Résultats définitifs de la campagne agricole 1997/1998 à 2002/2003. Récaputilatif des cultures industrielles et autres cultures.

[11] Yamaguchi T., Blumwald E. (2005). Developing salt-tolerant crop plants: challenges and opportunities. *Trends in Plant Science*.

[12] Food Agriculture Organization http://www.fao.org/ag/agl/agll/spush/.

[13] Munns R., Tester M. (2008). Mechanisms of salinity tolerance. *Annual Review of Plant Biology*.

[14] Levigneron A., Lopez F., Vansuyt G., Berthomieu P., Fourcroy P., Casse-Delbart F. (1995). Les plantes face au stress salin. *Cahiers Agricultures*.

[15] Hasegawa P. M., Bressan R. A., Zhu J.-K., Bohnert H. J. (2000). Plant cellular and molecular responses to high salinity. *Annual Review of Plant Biology*.

[16] Rengasamy P. (2002). Transient salinity and subsoil constraints to dryland farming in Australian sodic soils: an overview. *Australian Journal of Experimental Agriculture*.

[17] Klar A. E. (1984). *A Água No Sistema Solo-Planta-Atmosfer*.

[18] Dantas B. F., De Sáribeiro L., Aragão C. A. (2005). Physiological response of cowpea seeds to salinity stress. *Revista Brasileira De Sementes*.

[19] Murillo-Amador B., Troyo-Diéguez E., García-Hernández J. L. (2006). Effect of NaCl salinity in the genotypic variation of cowpea (*Vigna unguiculata*) during early vegetative growth. *Scientia Horticulturae*.

[20] Kajal G. P., Rao V. R. (2007). Effect of simulated water stress on the physiology of leaf senescence in three genotypes of cowpea (*Vigna unguiculata* (L.) Walp). *Indian Journal of Plant Physiology*.

[21] Chen C., Tao C., Peng H., Ding Y. (2007). Genetic analysis of salt stress responses in asparagus bean (*Vigna unguiculata* (L.) ssp. *sesquipedalis* Verdc.). *Journal of Heredity*.

[22] Hussein M. M., Balbaa L. K., Gaballah M. S. (2007). Developing a salt tolerant cowpea using alpha tocopherol. *Journal of Applied Sciences Research*.

[23] Tawfik K. M. (2008). Evaluating the use of rhizobacterin on cowpea plants grown under salt stress. *Research Journal of Agriculture and Biological Sciences*.

[24] Hall A. E., Frate C. A. (1996). *Blackeye Bean Production in California*.

[25] Serrano R., Mulet J. M., Rios G. (1999). A glimpse of the mechanisms of ion homeostasis during salt stress. *Journal of Experimental Botany*.

[26] Flowers T. J. (2004). Improving crop salt tolerance. *Journal of Experimental Botany*.

[27] Wahid A., Hameed M., Rasul E. (2004). Salt Injury symptom, changes in nutrient and pigment composition and yield characteristics of mungbean. *International Journal of Agriculture and Biology*.

[28] Saha P., Chatterjee P., Biswas A. K. (2010). NaCl pretreatment alleviates salt stress by enhancement of antioxidant defense system and osmolyte accumulation in mungbean (*Vigna radiata* l. wilczek). *Indian Journal of Experimental Biology*.

[29] Zhu J. K. (2001). Plant salt tolerance. *Trends in Plant Science*.

[30] Miller G., Suzuki N., Ciftci-Yilmaz S., Mittler R. (2010). Reactive oxygen species homeostasis and signalling during drought and salinity stresses. *Plant, Cell and Environment*.

[31] Hare P. D., Cress W. A., Van Staden J. (1998). Dissecting the roles of osmolyte accumulation during stress. *Plant, Cell and Environment*.

[32] Chen T. H. H., Murata N. (2002). Enhancement of tolerance of abiotic stress by metabolic engineering of betaines and other compatible solutes. *Current Opinion in Plant Biology*.

[33] Munns R. (2005). Genes and salt tolerance: bringing them together. *New Phytologist*.

[34] Roudani M. (1996). *Physiologie Comparée De Deux Espèces De Blé En Relation Avec Les Conditions De Nutrition. Métabolisme Racinaire En Milieu Salé*.

[35] Cissé N., Thiaw S., Ndiaye M. (1996). *Guide De La Production Du Niébé*.

[36] Hall A. E., Cisse N., Thiaw S. (2003). Development of cowpea cultivars and germplasm by the Bean/Cowpea CRSP. *Field Crops Research*.

[37] Badiane F. A., Gowda B. S., Cissé N., Diouf D., Sadio O., Timko M. P. (2012). Genetic relationship of cowpea (*Vigna unguiculata*) varieties from Senegal based on SSR markers. *Genetic and Molecular Research*.

[38] Viégas R. A., Melo A. R. B., Silveira J. A. G. (1999). Nitrate reductase activity and proline accumulation in cashew (*Anacardium occidentale* L.) in response to salt (NaCl) shock. *Revista Brasileira De Fisiologia Vegetal*.

[39] Côme D. (1968). Problèmes de terminologie posés par la germination et ses obstacles. *Bulletin De La Société Française De Physiologie Végétale*.

[40] Murashige T., Skoog F. (1962). A revised medium for rapid growth and bioassays, with tobacco tissue culture. *Physiologia Plantarum*.

[41] Arnon D. I. (1949). Cooper enzymes in isolated chloroplasts. *Plant Physiology*.

[42] Monneveux P., Nemmar M. (1986). Contribution à l'étude de la résistance à la sécheresse chez le blé tendre (*Triticum aestivum* L.) et chez le blé dur (*Triticum durum* Desf.): étude de l'accumulation de la proline au cours du cycle de développement. *Agronomie*.

[43] Development Core Team R. (2011). *A Language and Environment For Statistical Computing*.

[44] Bohnert H. J., Nelson D. E., Jensen R. G. (1995). Adaptations to environmental stresses. *Plant Cell*.

[45] Prado F. E., Boero C., Gallardo M., González J. A. (2000). Effect of NaCl on germination, growth, and soluble sugar content in *Chenopodium quinoa* Willd. seeds. *Botanical Bulletin of Academia Sinica*.

[46] Padilla E. G., Lopez Sanchez R. C., Eichler-Loebermann B., Fernandez-Pascual M., Barrero K. T., Martinez L. A. Salt stress affects on cowpea (*Vigna unguiculata* L. Walp) varieties at different growing stages.

[47] Botía P., Carvajal M., Cerdá A., Martínez V. (1998). Response of eight *Cucumis melo* cultivars to salinity during germination and early vegetative growth. *Agronomie*.

[48] Gill P. K., Sharma A. D., Singh P., Bhullar S. S. (2003). Changes in germination, growth and soluble sugar contents of *Sorghum bicolor* (L.) Moench seeds under various abiotic stresses. *Plant Growth Regulation*.

[49] Bohnert H. J., Jensen R. G., Flowers T. J., Yeo A. R. (1996). Metabolic engineering for increased salt tolerance—the next step. *Australian Journal of Plant Physiology*.

[50] IITA Cowpea. http://www.iita.org/cms/details/cereal_legumesaspx?=a86&2=63.

[51] Panet L., Holderbach l., Djemiah B. (1959). Influence des différentes concentrations en sel des eaux d'irrigation sur la croissance du riz. *Les AnnaLes De L'INRAT*.

[52] Kingsbury R., Epstein W. (1984). Selection for salt resistant spring wheat. *Crop Science*.

[53] Dvorak J., Ross K. (1986). Expression of tolerance of Na., K., Mg^2+^, CI-, and SO- ions and sea water in the amphiploid of Triticum aestivum x Elytrigia elongate. *Crop Science*.

[54] Yeo A. A., Flowers T. J., Stapes R. C., Toennienssen G. A. (1986). Mechanisms of salinity resistance in rice and their role as physiological criteria in plant breeding. *SalInity Tolerance In Plants. Strategies For Crop Improvement*.

[55] Mezni M. E., Bizid E., Harnza M. (1999). Effets de la salinité des eaux d'irrigation sur la survie et la croissance de trois cultivars de luzerne pérenne. *Fourrages*.

[56] Camara T. R., Willadino L., Torné A. M., Santos M. A. (2000). Efeito do estresse salino e da prolina exógena em calos de milho. *Revista Brasileira De Fisiologia Vegetal*.

[57] Murillo-Amador B., Troyo-Dieguez E., Jones H. G., Ayala-Chairez F., Tinoco-Ojanguren C. L., Lopez-Corte's A. (2000). Screening and classification of cowpea genotypes for salt tolerance during germination. *Phyton*.

[58] Brugnoli E., Björkman O. (1992). Growth of cotton under continuous salinity stress: influence on allocation pattern, stomatal and non-stomatal components of photosynthesis and dissipation of excess light energy. *Planta*.

[59] Bernstein N., Lauchli A., Silk W. K. (1993). Kinematics and dynamics of sorghum (*Sorghum bicolor* L.) leaf development at various Na/Ca salinities. I.Elongation growth. *Plant Physiology*.

[60] Evelin H., Kapoor R., Giri B. (2009). Arbuscular mycorrhizal fungi in alleviation of salt stress: a review. *Annals of Botany*.

[61] Gautheret R. (1981). *Effet du chlorure de sodium sur la croissance et l'alimentation minérale de citrus aurentium L. (bigaradier) et de l'hybride Poncirus trifoliata citru sinensis*.

[62] Pessarakli M. (1991). Dry matter yield, nitrogen-15 absorption, and water uptake by green bean under sodium chloride stress. *Crop Science*.

[63] Sanchez-blanco M. J., Bolarin M., Alarcon J. J., Torrecilas A. (1991). Salinity effect on water relations in *Lycoperscion esculentum* and its wild salt-tolerant relative species L.penneli. *Physiologia Plantarum*.

[64] LIyod J., Kriedemann P., Aspinall D. (1990). Contrasts between citrus species in reponse to salinization: an analysis of photosynthesis and water relation for different rootstock-scion combination. *Physiologia Plantarum*.

[65] Nieves M., Riuz D., Cedra A. Influence of rootstock-scion combination in Lemon trees salt tolerance.

[66] Chen C. T., Li C. C., Kao C. H. (1991). Senescence of rice leaves XXXI. Changes of chlorophyll, protein, and polyamine contents and ethylene production during senescence of a chlorophyll-deficient mutant. *Journal of Plant Growth Regulation*.

[67] Glenn C. W., Patten D. K., Drew M. C. (1993). Gas exchange and chlorophyll content of “Trif blue” rabbitey and “Sharp blue” southern highbush. Bluberry exposed to salinity and supplimental calcium. *Journal of the American Society For Horticultural Science*.

[68] Orcutt D. M., Nilsen E. T. (2000). *Physiology of Plants Under Stress*.

[69] Godde D., Lerner H. R., Dekker M. (1999). Adaptation of the photosynthetic apparatus to stress condition. *Plant Response to Environmental Stresses, From phytohormones to Genome Reorganization*.

[70] Ortega U., Duñabeitia M., Menendez S., Gonzalez-Murua C., Majada J. (2004). Effectiveness of mycorrhizal inoculation in the nursery on growth and water relations of Pinus radiata in different water regimes. *Tree Physiology*.

[71] Guettouche R. (1990). *Contribution à l'identification des caractères morphophysiologiques d'adaptation à la sécheresse chez le blé dur (Triticum durum Desf) [Ph.D. Thèse de diplôme d'agronomie approfondie]*.

[72] Tavakkoli E., Fatehi F., Coventry S., Rengasamy P., McDonald G. K. (2011). Additive effects of Na^+^ and Cl^−^ ions on barley growth under salinity stress. *Journal of Experimental Botany*.

[73] Steward C. R., Paleg L. G., Aspinal D. (1981). Proline accumulation: biochemical aspects. *The Physiology and Biochemistry of Drought Resistance in Plants*.

[74] Venekamp J. H. (1989). Regulation of cytosol acidity in plants under conditions of drought. *Physiologia Plantarum*.

[75] Silva J. V., De Lacerda C. F., Da Costa P. H. A., Filho J. E., Filho E. G., Prisco J. T. (2003). Physiological responses of NaCl stressed cowpea plants grown in nutrient solution supplemented with CaCl_2_. *Brasilian Journal of Plant Physiology*.

